# Genome sequencing of strains of the most prevalent clonal group of O1:K1:H7 *Escherichia coli* that causes neonatal meningitis in France

**DOI:** 10.1186/s12866-018-1376-4

**Published:** 2019-01-17

**Authors:** Guillaume Geslain, André Birgy, Sandrine Adiba, Mélanie Magnan, Céline Courroux, Corinne Levy, Robert Cohen, Philippe Bidet, Stéphane Bonacorsi

**Affiliations:** 1 IAME, UMR 1137, INSERM, Université Paris Diderot, Sorbonne Paris Cité, Paris, France; 2 Service de Microbiologie, Centre National de Référence Escherichia coli, Hôpital Robert-Debré, AP-HP, 48 boulevard Sérurier, 75019 Paris, France; 3 Institut de Biologie de l’Ecole Normale Supérieure, Ecole Normale Supérieure, PSL Research University Paris, Paris, France; 4 Association Clinique Thérapeutique Infantile du Val de Marne, Saint Maur des Fossés, France

**Keywords:** *Escherichia coli*, Meningitis, Newborns, Sequencing data analysis, Amoeba model

## Abstract

**Background:**

To describe the temporal dynamics, molecular characterization, clinical and ex vivo virulence of emerging O1:K1 neonatal meningitis *Escherichia coli* (NMEC) strains of Sequence Type complex (STc) 95 in France.

The national reference center collected NMEC strains and performed whole genome sequencing (WGS) of O1:K1 STc95 NMEC strains for phylogenetic and virulence genes content analysis. Data on the clinical and biological features of patients were also collected. Ex vivo virulence was assessed using the *Dictyostelium discoideum* amoeba model.

**Results:**

Among 250 NMEC strains collected between 1998 and 2015, 38 belonged to O1:K1 STc95. This clonal complex was the most frequently collected after 2004, representing up to 25% of NMEC strains in France. Phylogenetic analysis demonstrated that most (74%) belonged to a cluster designated D-1, characterized by the adhesin FimH30. There is no clinical data to suggest that this cluster is more pathogenic than its counterparts, although it is highly predominant and harbors a large repertoire of extraintestinal virulence factors, including a pS88-like plasmid. Ex vivo virulence model showed that this cluster was generally less virulent than STc95 reference strains of O45_S88_:H7 and O18:H7 serotypes. However, the model showed differences between several subclones, although they harbor the same known virulence determinants.

**Conclusions:**

The emerging clonal group O1:K1 STc95 of NMEC strains is mainly composed of a cluster with many virulence factors but of only moderate virulence. Whether its emergence is due to its ability to colonize the gut thanks to FimH30 or pS88-like plasmid remains to be determined.

**Electronic supplementary material:**

The online version of this article (10.1186/s12866-018-1376-4) contains supplementary material, which is available to authorized users.

## Background

*Escherichia coli* is a commensal of the gastrointestinal tract of vertebrates, including humans [1]. It is also involved in intestinal and extraintestinal infections [2]. In particular, *E. coli* is the most frequent bacteria involved in preterm meningitis and the second most frequent in newborn meningitis, causing a high rate of mortality or sequelae [3, 4].

Sequence types (ST) are grouped into clonal complexes by their similarity to a central allelic profile (genotype). Most *E. coli* strains responsible for neonatal meningitis belong to Sequence Type complex 95 (STc95) in the Warwick Multi-Locus Sequence Typing (MLST) scheme [5] and are mainly of serotypes O18:K1:H7, O1:K1, O7:K1, O83:K1, and the more recently reported O45_S88_:K1:H7 [6–8]. Complete sequencing of strain S88, representative of clone O45_S88_:K1:H7, has highlighted the presence of a 134 kb-plasmid, called pS88, encoding three iron capture systems (aerobactin, salmochelin, and SitACBD) and other putative extraintestinal virulence genes. The important role of this plasmid in the experimental pathogenicity of S88 has been demonstrated [8]. Plasmids similar to pS88 (pS88-like plasmid) have also been detected in other clonal groups responsible for meningitis, such as O18:K1, O1:K1 and O83:K1 [8].

Between 2001 and 2013, a French prospective national survey collected data for 325 children hospitalized with *E. coli* meningitis [5] and 141 *E. coli* isolates were sent to the national reference center (NRC) and studied. The highly pathogenic phylogenetic subgroup STc95 represented 56% of the 141 strains analyzed and among them, serogroup O1 (27.7%) was the most frequently identified during this period relative to serogroups O18 (19.1%) and O45_S88_ (11.3%).

Whole genome sequencing (WGS) of a large collection of strains belonging to STc95 was recently performed [9], but no O1:K1 neonatal meningitis strain belonging to this complex has been comprehensively characterized. We performed WGS of all the 38 O1:K1 STc95 strains isolated from 1998 to 2015 to gain insight into the pathophysiology of neonatal meningitis and their disease potential. Moreover, we analyzed the virulence of representative strains using the *Dictyostelium discoideum* amoeba model as previously used to evaluate *E. coli* extraintestinal pathogenic strains [10].

## Results

### Epidemiology

**Fig. 1:**
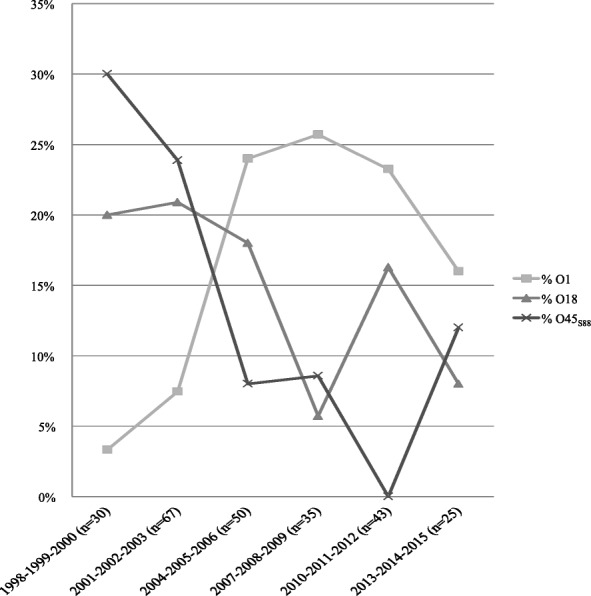
Percentage of STc95 isolates belonging to serogroups O1, O18, or O45_S88_ among all *E. coli* causing neonatal meningitis analyzed in the French reference center from 1998 to 2015 according to six different periods (*n* = 250)

We characterized the temporal dynamics of the three major STc95-serogroups that cause neonatal meningitis, O1, O18, and O45_S88_, by determining their respective rates between 1998 and 2015 (Fig. 1). These three serogroups represented 30 to 50% of the *E. coli* meningitis strains among the 250 NMEC collected by the NRC during the study period. Serotype O45_S88_:K1 largely predominated between 1998 and 2003, whereas O1:K1 isolates became the main serotype in STc95 NMEC in 2004 and thereafter. The 38 O1:K1 STc95 strains that were subjected to WGS represented up to 25% of *E. coli* meningitis strains received each year in the NRC. O18:K1 serotype is still present in the epidemiological landscape from 1998 to 2015 but never in the majority, with a percentage oscillating between 6 and 21%.

### Genotyping

**Fig. 2:**
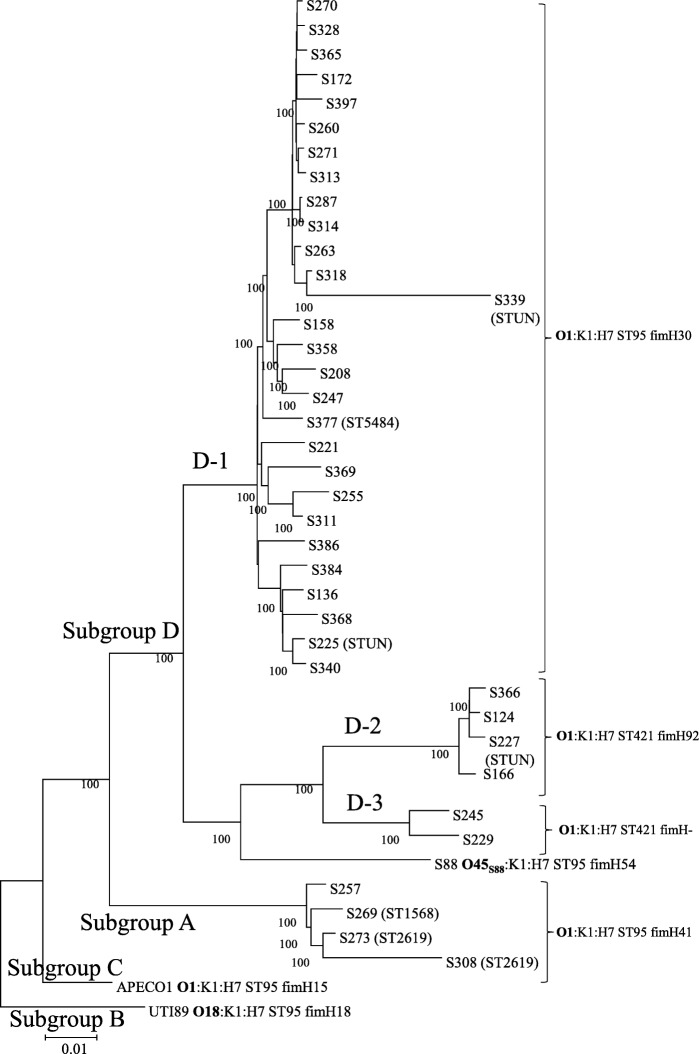
Phylogenetic analysis of STc95 O1:K1 neonatal meningitis *E. coli.* This phylogenetic tree of STc95 strains was based on 19,547 SNP sites identified by Illumina whole genome sequencing using the Neighbor-Joining method with 100 bootstrap replicates. Subgroup designations (A, B, C,D) are based on the classification of Gordon et al. [9]. Strains with names beginning with the letter S correspond to neonatal meningitis strains. APECO1, S88, and UTI89 were used as reference strains for comparison. The tree was rooted on UPEC strain CFT073. All NMEC strains are ST95 or ST421 except where indicated between brackets. Three strains carried unnamed STs (STUN). FimH alleles are also indicated

All our O1:K1 NMEC belonged to ST95, except 12 strains belonging to ST421, ST1568, ST2619, and ST5484 (and three strains harbored an unnamed ST). All these STs differ from ST95 by one allele and are therefore considered to be part of STc95 (Fig. 2).

A WGS phylogenetic tree, based on the distribution of core-genome SNPs, clustered NMEC O1:K1 strains into two major subgroups, A and D. These are two of the five subgroups among STc95 *E. coli* described by Gordon et al. [9]. Within subgroup D, we identified three clusters called D-1, D-2 and D-3, with most NMEC isolates (74%) belonging to cluster D-1.

Strain S88, representative of the O45_S88_:K1 clonal group, appears to be more closely related to clusters D-2 and D-3 than the major cluster, D-1, whereas the representative strain of APEC O1:K1 seems to be paradoxically more distantly related (subgroup C) (Fig. 2).

### Virulence genotypes

The distribution of extraintestinal virulence factors identified by WGS in subgroup A and clusters D-1 and D-2/D-3 is depicted in Table 1. Cluster D-1 possessed the most ExPEC genetic determinants, as almost all isolates harbored all iron transport systems, bacteriocins, and other ColV plasmid-related genes found in pS88-like plasmids. Conversely, toxin genes were more prevalent among subgroup A and clusters D-2/D-3. Several factors involved in the pathophysiology of meningitis, such as Cnf1, Sfa/foc, and IbeA, were absent from our O1:K1 strains.

**Table 1:** Distribution of putative virulence factors and bacteriocins detected in silico among O1:K1 NMEC strains

Genes	Rate (%) among STc95 subgroups/clusters			
	A (*n* = 4)	D-1 (*n* = 28)	D-2/D-3^b^ (*n* = 6)	Total (*N* = 38)
Adhesins				
*papGII*	100	100	100	100
*iha*	0	3.6	100	18.4
Iron transport				
*iucC, iutA*	25	92.6	100	86.8
*fyuA, irp*	100	100	100	100
*iroN, D*	50	100	100	94.7
*chuA*	100	100	100	100
*sitA*	75	100	100	97.4
Bacteriocins				
*cvaA, C*	50	100	0	78.9
*cvi*	50	100	100	94.7
*cia*	0	100	0	73.7
*imm*	50	100	0	78.9
*mchB, C*	0	3.6	0	2.6
*mchF*	50	100	0	78.9
ColV plasmid related				
*tsh*	25	0	0	2.6
*etsC*	50	100	0	78.9
*ompTp*^*a*^	50	100	0	78.9
*hlyF*	50	100	0	78.9
*mig-14*	50	100	0	78.9
*iss*^*a*^	50	100	0	78.9
*traj*	0	100	0	73.7
*eitB*	25	0	0	2.6
Toxin				
*hlyC*	0	0	100	15.8
*clbB, N*	100	0	0	10.5
*sat*	0	0	100	15.8
*vat*	75	92.6	100	92.1
*senB*	50	0	0	5.3
Others				
*epeA*	0	3.6	0	2.6
*ireA*	100	100	100	100
*gad*	100	100	100	100

^a^: plasmidic genes

^b^: D-2 and D-3 clusters have been merged since they harbor identical genetic determinants except for *fimH* (Fig. 2)

Only virulence genes present at least in one strain are indicated, other genes not detected are not shown [see Additional file 4]

FimH is an adhesin of type I that is involved in the adhesion of endothelial cells preceding the invasion of cerebral spinal fluid (CSF) [11]. The distribution of the various alleles of FimH or the absence of this gene is in accordance with the genetic subgroups and clusters (Fig. 2). The major allele, FimH30, differs from those of the O45_S88_:K1 (S88) and O18:K1 (UTI89) reference strains, FimH54 and FimH18, respectively.

### Antimicrobial resistance

No extended spectrum beta-lactamase or carbapenemase was found among these meningitis isolates. Seventeen strains (45%) produced a penicillinase, all harboring *bla*TEM-1 except one strain harboring *bla*TEM-84. Alleles of *bla*TEM-1 were *bla*TEM-1-A (*n* = 1), *bla*TEM-1-B (*n* = 8) and *bla*TEM-1-C (*n* = 7). Allele *bla*TEM-1-B was associated with genes encoding resistance to streptomycin (*strAB*) and sulphonamide (*sul2* and *dfrA5*) in all cases but one. Antimicrobial resistance was not associated with a particular WGS subgroup or with pS88-associated genes. Thirteen isolates were devoid of any antimicrobial resistance gene.

### Clinical and biological features of patients infected by O1:K1 STc95 *E. coli* strains

We compared patients infected by strains belonging to the main epidemiological cluster D-1 to those infected by strains belonging to other clusters. Patients infected by cluster D-1 were younger, less mature, and of lower birth weight, although the differences were not statistically significant (Table 2). Although cluster D-1 was largely predominant, no clinical data support higher pathogenicity of this cluster relative to its counterparts. Median CSF glucose was lower in patients infected by cluster D-1 and median CSF protein higher than for the other subgoups, as well as the number of cells in CSF, which was significantly higher (*p* = 0.02) (Table 2).

**Table 2:** Clinical and biological data of the patients according to the *E. coli* O1K1 subgroups

Clinical and biological characteristics	Prevalence of characteristics among the following subgroups:		
	D-1 (*n* = 28)	A + D-2 + D-3^a^ (*n* = 10)	*p* value*
Gender (M/W)	12/14 (26)	5/3 (8)	0.69
Median of term (AW)	37 [29–41] (20)	39 [34–41] (6)	0.18
Median of birth weight (grams)	3160 [950–3630] (19)	3555 [2260–4240] (6)	0.13
Median of age at the LP (days)	13.5 [1–136] (28)	28 [3–145] (9)	0.25
Median of CSF glucose (mmol/l)	0.4 [0–3.6] (12)	1.9 [0.4–3.4] (4)	0.22
Median of CSF protein (g/l)	2.2 [0.7–5.1] (12)	1.6 [1.1–10] (3)	1
Median of number of cells in CSF (/mm^3^)	9300 [800–60,000] (12)	1320 [60–3158] (4)	0.02
Median of neutrophil polynuclear % in CSF	85 [72–95] (9)	92 [90–95] (3)	0.48
% of Positive blood culture	54 (11)	50 (4)	1

Values in parentheses correspond to the number of patients for whom information as available

Values in brackets correspond to minimum and maximum

*Fisher’s exact test was used for comparison of proportion and categorical variables

*Mann-Whitney test was used for comparison of median and continuous variables

^a^Subgroup A and clusters D-2 and D-3 have been merged for the statistical analysis

### Ex vivo model with amoeba Dictyostelium discoideum

Grazing scores of the amoeba virulence assay are not shown [see Additional file 1]. The ex vivo model with amoeba *D. discoideum* did not reveal clear associations between phylogenetic subgroups and grazing resistance. Generally, the O1:K1 strains were less virulent in this model than representative strains of the O45_S88_:K1 and O18:K1 serotypes and the virulent control strains. However, the different subclones were not equally virulent. Indeed, among the D-1 strains, the subclone containing strains S158, S208, and S358 killed *D. discoideum* more efficiently (mean score 0.06) than the subclone containing strains S136, S368, S384, and S386 (mean score 0.65).

We tested a representative strain of cluster D-1, S172, cured of its plasmid, and a transconjugant of J53 in our model to determine whether plasmid pS88-like may play a role in the virulence of O1:K1 D-1 strains in our model. The grazing score was clearly affected, with a loss of virulence for S172△pS172, while J53pS172 gained virulence [see Additional file 1].

## Discussion

The clonal complex STc95 is one of the major *E. coli* lineages that causes human extraintestinal infections. The extraintestinal virulence of STc95 *E. coli* is exemplified by their ability to cause neonatal meningitis. In France, this group represents 56% of the neonatal meningitis strains collected by the national reference center [5]. WGS performed on several hundred STc95 strains from various parts of the world has provided a comprehensive view of the genetic organization of this clonal complex, as well as its geographical distribution and temporal dynamics [9]. However, among the 500 strains investigated in this study, only two were isolated from the CSF of neonates (O18:K1 and O45_S88_:K1). During the last 18 years, there has been a change in the relative frequency of major serotypes in STc95 NMEC, with a significant increase of O1:K1:H7 strains that were characterized by WGS.

Among the three major subgroups (A, C, D) that contain O1:H7 strains, described by Gordon et al. [9], strains responsible for meningitis, collected throughout France, belonged exclusively to subgroups A and D. The absence of O1:H7 subgroup C strains in our collection may be related to their lower potential to cause disease relative to other subgroups or their specific geographical distribution, as they were only found in Australia in the world-wide collection [9]. O1:K1:H7 meningitis strains of our study were more frequently found in subgroup D than in subgroup A (*n* = 34 versus *n* = 4). These two subgroups were present in all continents studied (USA, Europe, and Australia) with a similar repartition (*n* = 8 and *n* = 12 for subgroup A and D, respectively [9]. Subgroup D which predominates among our collection may have a greater potential to cause neonatal meningitis than subgroup A.

Phylogenetic analysis allowed us to distinguish three clusters within subgroup D, called D-1 (*n* = 28), D-2 (*n* = 4), and D-3 (*n* = 2). The closely related clusters D-2 and D-3 are minor relative to cluster D-1 and carry a different ST (ST421) and a different FimH (92) or no FimH gene (D-3). Cluster D-1, which carries FimH30, may represent a group of strains with a high capacity to induce neonatal meningitis. It also carries genetic determinants characteristic of the extraintestinal virulence plasmid pS88, which are absent from clusters D-2 and D-3, and rarely present in subgroup A. This plasmid may be key to the virulence of this group, as shown for the recently described clone O45_S88_:K1:H7 [8]. The representative strain of clone O45_S88_:K1:H7, S88, which carries the pS88 plasmid, appears to be closely related to D-2 and D-3 strains. Thus, it is possible that clone O45_S88_:K1:H7 was derived from D-2/D-3 strains, after switching its O antigen gene cluster and acquiring the pS88 plasmid.

Analysis of the clinical features of infected neonates did not provide evidence that cluster D-1 is more virulent than D-2/D-3, despite the large number of strains of cluster D1 and the presence of plasmid pS88. However, this cluster appeared to elicit a larger inflammatory response.

Several factors known to be involved in the pathophysiology of neonatal meningitis were completely absent from our collection, i.e. *ibeA*, *cnf1*, and *sfa.* This highlights the variation in the virulence factor repertoire that leads to acute bacteremia and crossing of the blood brain barrier, the two major steps of this infection. Several studies have attempted to define a potential NMEC pathotype [12, 13]. For example, Wijetunge et al. compared 26 genes encoding virulence factors between 53 NMEC strains and 48 fecal strains of healthy individuals and found that the combination of K1 capsule, aerobactin siderophore, vacuolating cytotoxin (Vat), and the iron-binding protein (Sit) are typical traits of NMEC [13]. Among toxins, only vacuolating cytotoxin was present in almost all O1:K1 NMEC strains, irrespective of genetic subgroup, reinforcing the potential role of this toxin in the physiopathology of neonatal meningitis.

We assessed the experimental virulence of O1:K1:H7 and representative and control strains in the amoeba *D. discoideum* model, previously used to assess *E. coli* resistance to phagocytosis [10]. Our aim was to analyze possible fine differences between meningitis-causing clones and not to simulate the global pathophysiology of meningitis. This model avoids the use of animals and is of interest because it is performed at a low temperature (22 °C). At this temperature, the K1 capsule, the major virulence factor of NMEC, which may mask other bacterial traits involved in virulence, is inactivated [14]. Its inactivation may facilitate analysis of the potential role of other factors. Indeed, we assessed the production of the capsule of our O1:K1 strains by the agglutination test after culture at 22 °C and 35 °C and found that the K1 capsule was undetectable at 22 °C, but present at 35 °C (data not shown). Generally, the O1:K1 strains appeared to be less virulent than representative strains of O45_S88_:H7 and O18:H7 serotypes and the control virulent strains. However, we found that they were not equally virulent upon analysis of each subclone. Among the D-1 strains, closely related subclones, with an identical repertoire of virulence genes behaved differently in the *D. discoideum* model. This highlights the complexity of the regulation of virulence and the involvement of various factors that are currently not known. Moreover, since most virulence factors implicated in human pathogeny are more efficient at 37 °C, it is also likely that the amoeba model underestimates their role. Nevertheless, we were able to show that the pS88-like plasmid plays a role in the resistance against phagocytosis using isogenic strains, thus complementing its previously described role in survival to bactericidal activity of serum [8].

Another notable difference between the O1:K1 clusters is the adhesin FimH. It binds specifically to D-mannose residues attached to the surface of glycoproteins that line vaginal, perineal, and bladder cells, as well as enterocytes [15]. FimH is expressed by more than 95% of *E. coli* and genetic variation can change its tropism [16]. It also plays an important role in the adhesion and invasion of endothelial cells of brain capillaries by NMEC in humans [17]. However it may be dispensable, since we found two meningitis-causing strains, S229 and S245, with no *fim*H gene (this was confirmed by *fimH*-specific PCR, data not shown).

The strains of NMEC O1:K1:H7 described here, carry mostly the fimH30 allele, whereas the other main serotypes responsible for meningitis carry the FimH54 (O45:K1:H7) and FimH18 (O18:K1:H7) alleles. Of note, most strains of the multi-resistant epidemic clonal group of *E. coli* ST131 O25b:H4, found throughout the world, carry FimH30 [18]. A recent study has shown that this clone display a greater adherence to CaCo2 enterocytes compared to other ESBL-producing *E. coli* isolates, although the specific role of FimH30 was not assessed [19]. It is possible that FimH30 allele confer an advantage to strains of the D-1 cluster for gut colonization, thus aiding their expansion.

Antimicrobial resistance was limited to the production of penicillinase (encoded by *bla*TEM-1 except for one strain), resistance to streptomycin, tetracyclin and sulphonamid and about one third of strains were devoid of any acquired resistance mechanism. We found no association between resistance and a particular WGS-subgroup or with the presence of pS88 virulence factors, suggesting that virulence and resistance genes are harbored by different plasmids or genomic islands such as Integrative and Conjugative Elements (ICE).

## Conclusions

This work describes the recent dominance of the meningitis-causing *E. coli* O1:K1:H7, belonging to the highly virulent STc95, in France. We used WGS to describe its genetic diversity and found the predominance of a major cluster (D-1), characterized by the presence of the pS88-like plasmid. Clinical data and an ex vivo model failed to show higher pathogenicity of strains belonging to this major cluster D-1. Whether its high prevalence results from a high capacity to colonize the gut, due to adhesion through the FimH30 allele and/or the presence of the pS88-like plasmid, are yet to be determined.

## Methods

### *E. coli* isolates and clinical data

Between January 1, 1998 and December 31, 2015, 250 *E. coli* strains responsible for neonatal meningitis in France were collected by the *E. coli* national reference center (NRC). All strains were subjected to serogroup and *svg* PCR [20] [see Additional file 2]. By this method, 38 O1:K1 *E. coli* of STc95 were identified among these isolates and included in the study for WGS.

Clinical and biological data (age and sex of the patient, birth term, birth weight, date of onset of the infection, blood culture, and biological characteristics of the lumbar puncture) were collected.

### Whole-genome sequencing

The whole genomes of the 38 *E. coli* isolates were sequenced. The Nextera XT kit (Illumina) was used to prepare libraries. Sequencing was performed on a MiSeq instrument using reagent kit V3 600 cycles (Illumina technology). Sequences were analyzed using the Center of Genomic Epidemiology (CGE) website (www.genomicepidemiology.org). Genes were annotated using Rast® software (version 2.0), and the sequences aligned and compared using BioEdit® Sequence Alignment Editor (V7.2.5).

### Sequencing data quality

The SPAdes assembler was used to construct assemblies. Contigs < 500 bp were removed. The quality of the de novo assemblies was estimated using standard metrics [see Additional file 3]. Mean N50 were of 127,597 and mean N75 were of 70,794. Finally, reads used to construct the assemblies were remapped against the assembly contigs to visualize coverage. Mean coverage was of 37.

### STc95 and subgroups

The CGE website was used to confirm the ST of the strains using the MLST tool [21]. In silico PCR was performed to determine STc95 subgroups (A,B,C,D and E), as described by Gordon et al. [9].

### Phylogeny

SNP (single nucleotide polymorphism) calling was performed with CSI Phylogeny (version 1.4) available on CGE web site (https://cge.cbs.dtu.dk/services/CSIPhylogeny/). CFT073 strain (O6:K5:H1) was used as outgroup in order to restrict our analysis to core genome SNPs. A phylogenetic tree, based on concatenated alignment of the 19,547 SNPs identified, was constructed by the Neighbor-Joining method available on MEGA software (version 3.1) with 100 bootstrap replicates [22].

### Detection of putative virulence genes and genes encoding antimicrobial resistance

Genes encoding antimicrobial resistance and putative virulence factors were identified using the Resfinder and VirulenceFinder tools available on CGE website [23]. Moreover, 166 genes [Additional file 4] were also assessed by local blast analysis using the NCBI blast tool (Blast+ version 2.2.31). Genes with an alignment coverage higher than 90% and a homology > 90% were considered to be present. Strains harboring > 90% of plasmid pS88 sequence were considered as harboring a pS88-like plasmid. The FimH allele nomenclature of Sokurenko et al. was used [16].

### Ex vivo model with Amoeba Dictyostelium discoideum

The virulence of neonatal meningitis *E. coli* (NMEC) strains O1:K1 was assessed by their resistance to phagocytosis in an amoeba ex vivo model as described by Adiba et al. [10]. We assessed 26 *E. coli* strains [Additional file 1], including 19 representative of our collection and the reference meningitis strains C5 (O18:K1) and S88 (O45_S88_:K1) [7, 8]. *E. coli* REL606 was used as a positive control for phagocytosis and the pathogenic strain *E. coli* 536 as a negative control [24]. We also evaluated the role of the pS88-like plasmid in virulence and resistance to phagocytosis by curing one natural isolate (S172) of its plasmid (S172ΔpS172) and generating a transconjugant of the avirulent strain J53 (J53p172), as previously described [8].

The previously described amoebic phagocytosis protocol [10] was used, except that we used three amoeba population sizes (10^3^, 10^4^, 10^5^ cells) for each bacterial population. Each strain was tested in triplicate. Results were interpreted in terms of *D. discoideum* grazing. Strains not able to resist amoebic phagocytosis were considered to be grazing sensitive (GS) (and thus less virulent) and strains resistant to amoebic phagocytosis, grazing resistant (GR) (and thus more virulent). A grazing score was calculated for each strain, corresponding to the mean of each series of replicates: 0 for GR and 1 for GS.

### Statistical analysis

Categorical variables were compared using the Fischer exact-test. Continuous variables were expressed as the mean ± SD and compared using the Mann-Whitney test. A *p* value < 0.05 was considered statistically significant.

## Additional files

Additional file 1: ex vivo amoeba model outcomes for several representatives of O1:K1 STc95 *E. coli* meningitis strains; description: grazing scores for representative strains. (DOCX 15 kb)

Additional file 2: Primers of serotype PCR; description: list of primers and sequences used for serotype PCR. (DOCX 17 kb)

Additional file 3: Sequencing data information; description: N50, N75, Minimum contig Length, Maximum contig Length, Average contig Length, Total contig length, Mean coverage, Number of reads, Number of contigs for all O1:K1 *E. coli* isolates. (DOCX 22 kb)

Additional file 4: 166 genes searched by local blast analysis using the NCBI blast tool; description: list of genes searched in the strains. (DOCX 15 kb)
