# Supplementary material for: Genome sequencing of strains of the most prevalent clonal group of O1:K1:H7 Escherichia coli that causes neonatal meningitis in France
Source: BMC Microbiol. 2019 Jan 17;19:17. doi: 10.1186/s12866-018-1376-4 (PMC6337857; doi:10.1186/s12866-018-1376-4)
Supplement: Supplementary file 1 — ex vivo amoeba model outcomes for several representatives of O1:K1 STc95 E. coli meningitis strains; description: grazing scores for representative strains. (DOCX 15 kb) [file 12866_2018_1376_MOESM1_ESM.docx]

Additional file 1: *ex vivo* amoeba model outcomes for several representatives of O1:K1 STc95 *E. coli* meningitis strains

| **Characteristic** | **Strain** | **Serotype** | **Clonal subgroup** | **Grazing score** |
| --- | --- | --- | --- | --- |
| NMEC | S257 | O1:K1 | A | 1.0 |
| NMEC | S308 | O1:K1 | A | 0.2 |
| NMEC | S136 | O1:K1 | D-1 | 0.9 |
| NMEC | S368 | O1:K1 | D-1 | 0.6 |
| NMEC | S384 | O1:K1 | D-1 | 0.7 |
| NMEC | S158 | O1:K1 | D-1 | 0.0 |
| NMEC | S208 | O1:K1 | D-1 | 0.1 |
| NMEC | S358 | O1:K1 | D-1 | 0.1 |
| NMEC | S172 | O1:K1 | D-1 | 0.4 |
| NMEC | S260 | O1:K1 | D-1 | 0.3 |
| NMEC | S270 | O1:K1 | D-1 | 0.3 |
| NMEC | S311 | O1:K1 | D-1 | 0.6 |
| NMEC | S377 | O1:K1 | D-1 | 0.0 |
| NMEC | S386 | O1:K1 | D-1 | 0.4 |
| NMEC | S124 | O1:K1 | D-2 | 0.0 |
| NMEC | S227 | O1:K1 | D-2 | 0.3 |
| NMEC | S366 | O1:K1 | D-2 | 0.4 |
| NMEC | S229 | O1:K1 | D-3 | 0.0 |
| NMEC | S245 | O1:K1 | D-3 | 0.2 |
| NMEC | S88 | O45_S88_:K1 |  | 0.0 |
| NMEC | C5 | O18:K1 |  | 0.0 |
| avirulent | J53 | O16:K- |  | 1.0 |
| variant | S172 Δ pS172 | O1:K1 | D-1 | 0.7 |
| transconjugant | J53pS172 | O16:K- |  | 0.7 |
| avirulent control | REL606 | O7:K- |  | 1.0 |
| UPEC virulent control | 536 | O6:K15 |  | 0.0 |
