# Supplementary material for: Genome sequencing of strains of the most prevalent clonal group of O1:K1:H7 Escherichia coli that causes neonatal meningitis in France
Source: BMC Microbiol. 2019 Jan 17;19:17. doi: 10.1186/s12866-018-1376-4 (PMC6337857; doi:10.1186/s12866-018-1376-4)
Supplement: Supplementary file 2 — Primers of serotype PCR; description: list of primers and sequences used for serotype PCR. (DOCX 17 kb) [file 12866_2018_1376_MOESM2_ESM.docx]

Additional file 2: primers of serotype PCR

| **Primers** | **Sequence 5'- 3'** | **Gene** |
| --- | --- | --- |
| wzy-O1-F | GCAATTGCAGTCACTATGTGC | *O1* |
| wzy-O1-R | AGGCGCTGTAATGAAACAAAA |  |
| wzy-O2-F | CAAGCATTGGAAATGAAAGGA | *O2* |
| wzy-O2-R | CACATTAATCACTCCAAAGACCA |  |
| wzy-O4-F | TTTTAACACACAGGCGACAGG | *O4* |
| wzy-O4-R | TGCTCATTAACCATGCAGTCA |  |
| wzy-O6-F | CGGCATGGGAGTATCATATCTT | *O6* |
| wzy-O6-R | GTGTATGAGGCCAAATGGCTA |  |
| wzy-O7-F | TCAAACAGTTTCACACGTTGC | *O8* |
| wzy-O7-R | ATGGATTCACATTCCATGAGC |  |
| wzy-O16-F | AGAATTTGTGTGGGTGGGTTT | *O16* |
| wzy-O16-R | ATTTTCTGGGCCTTAAGAAATTG |  |
| wzy-O18-F | GATCATTGTCATCCCAATTTCA | *O18* |
| wzy-O18-R | GGTCATTCTCAGCCATACGTG |  |
| wzyS88.F | GGTATCGTTCACATCGCTTA | *O45_S88_* |
| wzyS88.R | GAGAAAATACTCGGTTCGGC |  |
| wzy-O83-F | ATTTACTGGAGGATGGGCAAT | *O83* |
| wzy-O83-R | TGAACTACTGTCTCCGCCATT |  |
| neu-K1-600-F | AGAGGAGGAGTTCCCAATTAGC | *K1* |
| neu-K1-600-R | CACATATGATGCGGTTATGGTT |  |
| uidA-750-F | GCCTCTTCGCTGTACAGTTCTT | *uidA* |
| uidA-750-R | ACGTATCACCGTTTGTGTGAAC |  |
