# Supplementary material for: Genome sequencing of strains of the most prevalent clonal group of O1:K1:H7 Escherichia coli that causes neonatal meningitis in France
Source: BMC Microbiol. 2019 Jan 17;19:17. doi: 10.1186/s12866-018-1376-4 (PMC6337857; doi:10.1186/s12866-018-1376-4)
Supplement: Supplementary file 3 — Sequencing data information; description: N50, N75, Minimum contig Length, Maximum contig Length, Average contig Length, Total contig length, Mean coverage, Number of reads, Number of contigs for all O1:K1 E. coli isolates. (DOCX 22 kb) [file 12866_2018_1376_MOESM3_ESM.docx]

Additional file 3: Sequencing data information

| Isolate | N50 | N75 | Minimum contig Length | Maximum contig Length | Average contig Length | Total contig length | Mean coverage | Number of reads | Number  of contigs |
| --- | --- | --- | --- | --- | --- | --- | --- | --- | --- |
| S124 | 204 025 | 103 782 | 500 | 722 969 | 36 569 | 5 046 533 | 48 | 1 186 300 | 138 |
| S136 | 100 149 | 45 759 | 510 | 291 091 | 35 121 | 5 057 386 | 34 | 838 530 | 144 |
| S158 | 174 681 | 107 151 | 510 | 575 487 | 38 255 | 5 240 931 | 42 | 1 014 848 | 137 |
| S166 | 139 414 | 90 751 | 501 | 283 454 | 47 506 | 5 273 128 | 41 | 1 053 068 | 111 |
| S172 | 58 608 | 33 291 | 505 | 218 257 | 21 661 | 5 025 263 | 24 | 516 682 | 232 |
| S208 | 47 380 | 24 719 | 508 | 195 409 | 17 870 | 5 110 737 | 26 | 613 156 | 286 |
| S221 | 102 168 | 51 445 | 528 | 447 864 | 38 065 | 5 138 841 | 33 | 804 902 | 135 |
| S225 | 100 148 | 51 013 | 501 | 254 996 | 39 848 | 4 941 147 | 32 | 775 476 | 124 |
| S227 | 78 208 | 43 325 | 500 | 220 342 | 30 462 | 4 934 827 | 31 | 764 980 | 162 |
| S229 | 95 752 | 49 034 | 531 | 251 699 | 29 035 | 5 168 199 | 32 | 809 436 | 178 |
| S245 | 102 706 | 59 586 | 611 | 327 132 | 48 704 | 4 967 802 | 33 | 798 158 | 102 |
| S247 | 83 630 | 42 267 | 567 | 357 469 | 34 365 | 5 086 044 | 30 | 752 784 | 148 |
| S255 | 47 022 | 21 808 | 516 | 164 102 | 18 436 | 5 328 102 | 25 | 605 958 | 289 |
| S257 | 147 638 | 102 118 | 522 | 681 972 | 71 784 | 5 168 429 | 41 | 995 588 | 72 |
| S260 | 175 663 | 98 032 | 507 | 588 286 | 45 401 | 5 084 937 | 44 | 1 075 782 | 112 |
| S263 | 199 480 | 109 997 | 580 | 595 869 | 57 826 | 5 088 722 | 47 | 1 167 942 | 88 |
| S269 | 30 862 | 18 673 | 508 | 103 640 | 14 487 | 5 229 648 | 23 | 585 348 | 361 |
| S270 | 140 556 | 86 649 | 520 | 425 785 | 49 585 | 5 206 415 | 39 | 992 896 | 105 |
| S271 | 168 750 | 75 532 | 500 | 586 988 | 53 583 | 5 090 404 | 41 | 997 772 | 95 |
| S273 | 108 635 | 61 413 | 511 | 499 650 | 26 472 | 5 268 027 | 36 | 922 948 | 199 |
| S287 | 181 291 | 113 388 | 662 | 450 255 | 62 442 | 5 057 791 | 48 | 1 131 234 | 81 |
| S308 | 6 196 | 3 292 | 511 | 37 386 | 3 890 | 5 158 325 | 16 | 401 402 | 1 326 |
| S311 | 124 345 | 74 557 | 527 | 387 417 | 41 647 | 5 247 533 | 43 | 1 100 986 | 126 |
| S313 | 120 798 | 69 339 | 558 | 389 725 | 51 382 | 5 086 798 | 40 | 1 038 204 | 99 |
| S314 | 135 152 | 90 479 | 635 | 387 821 | 53 207 | 5 054 665 | 41 | 1 058 930 | 95 |
| S318 | 71 650 | 31 931 | 604 | 303 544 | 27 904 | 5 134 268 | 31 | 790 110 | 184 |
| S328 | 185 357 | 116 623 | 545 | 586 993 | 69 945 | 5 105 972 | 47 | 1 180 128 | 73 |
| S339 | 7 581 | 3 973 | 505 | 36 692 | 4 301 | 4 941 371 | 19 | 409 238 | 1 149 |
| S340 | 183 317 | 113260 | 558 | 864 321 | 70 004 | 5 040 275 | 45 | 1 088 268 | 72 |
| S358 | 117 008 | 73 439 | 525 | 301 028 | 41 432 | 5 096 093 | 38 | 973 578 | 123 |
| S365 | 141 322 | 74 685 | 500 | 3 877 001 | 41 077 | 5 216 716 | 45 | 1 258 108 | 127 |
| S366 | 131 351 | 100 921 | 500 | 465 127 | 55 664 | 4 954 067 | 38 | 982 900 | 89 |
| S368 | 66 602 | 39 321 | 513 | 174 742 | 28 083 | 4 970 668 | 31 | 785 588 | 177 |
| S369 | 182 126 | 99 993 | 581 | 416 966 | 66 500 | 5 053 998 | 45 | 1 164 596 | 76 |
| S377 | 264 263 | 129 527 | 559 | 446 843 | 65 555 | 5 047 738 | 57 | 1 419 566 | 77 |
| S384 | 268 286 | 113 100 | 558 | 866 212 | 82 422 | 5 110 152 | 43 | 1 073 626 | 62 |
| S386 | 215 603 | 132 924 | 632 | 571 721 | 67 728 | 5 147 298 | 53 | 1 343 380 | 76 |
| S397 | 64 960 | 33 071 | 502 | 144 528 | 24 124 | 5 065 979 | 28 | 682 844 | 210 |
