# Supplementary material for: Genome sequencing of strains of the most prevalent clonal group of O1:K1:H7 Escherichia coli that causes neonatal meningitis in France
Source: BMC Microbiol. 2019 Jan 17;19:17. doi: 10.1186/s12866-018-1376-4 (PMC6337857; doi:10.1186/s12866-018-1376-4)
Supplement: Supplementary file 4 — 166 genes searched by local blast analysis using the NCBI blast tool; description: list of genes searched in the strains. (DOCX 15 kb) [file 12866_2018_1376_MOESM4_ESM.docx]

Additional file 4: 166 genes searched by local blast analysis using the NCBI blast tool

| *papC* | *cnf1* | *espJ* | *agg3B* | *perA* | *eae-alpha2* |
| --- | --- | --- | --- | --- | --- |
| *papGI* | *hek* | *espP* | *agg3C* | *pet* | *eae-beta1* |
| *papGI-2* | *ibeA* | *etpD* | *agg3D* | *pic:1* | *eae-beta2* |
| *papGII* | *hlyC* | *f17A* | *agg4A* | *rpeA* | *eae-gamma1* |
| *papGIII* | *clbB* | *f17G* | *agg4B* | *senB* | *eae-gamma2* |
| *iucC* | *clbN* | *fanA* | *agg4C* | *sepA* | *eae-delta* |
| *iutA* | *sat* | *fasA* | *agg4D* | *sigA* | *eae-epsilon* |
| *fyuA* | *vat* | *fedA* | *agg5A* | *sta1* | *eae-zeta* |
| *irp2* | *mchB* | *fedF* | *aggA* | *stb* | *eae-eta* |
| *iroN* | *mchC* | *fim41a* | *aggB* | *stx2A-a* | *eae-theta* |
| *iroD* | *mchF* | *gad* | *aggC* | *stx2B-a* | *eae-iota* |
| *chuA* | *mcmA* | *ehxA* | *aggD* | *stx2A-b* | *eae-kappa* |
| *TSP-E4C2* | *astA* | *hlyE* | *aggR* | *stx2B-b* | *eae-lambda* |
| *yja* | *bfpA* | *iha* | *capU* | *stx2A-c* | *eae-mu* |
| *svg* | *cba* | *ipaD* | *eilA* | *stx2B-c* | *eae-nu* |
| *cvaA* | *ccI* | *ipaH9.8* | *ORF3* | *stx2A-d* | *eae-xi* |
| *cvi* | *cdtB* | *ireA* | *ORF4* | *stx2B-d* |  |
| *cvaC* | *celb* | *K88ab* | *aafA* | *stx2A-e* |  |
| *cia* | *cfaC* | *katP* | *aafB* | *stx2B-e* |  |
| *imm* | *cif* | *lngA* | *aafC* | *stx2A-f* |  |
| *shiF* | *cma* | *lpfA* | *aafD* | *stx2B-f* |  |
| *etsC* | *cofA* | *ltcA* | *aaiC* | *stx2A-g* |  |
| *ompTp* | *eatA* | *subA* | *aap* | *stx2B-g* |  |
| *hlyFp* | *efa1* | *saa* | *aar* | *stx1A-a* |  |
| *mig-14p* | *epeA* | *tccP* | *aatA* | *stx1B-a* |  |
| *issp* | *espA* | *tir* | *air* | *stx1A-c* |  |
| *sitAp* | *espB* | *toxB* | *nfaE* | *stx1B-c* |  |
| *traJp* | *espC* | *tsh* | *nleA* | *stx1A-d* |  |
| *sfaS* | *espF* | *virF* | *nleB* | *stx1B-d* |  |
| *focD* | *espI* | *agg3A* | *nleC* | *eae-alpha1* |  |
